# Laparoscopic and robot-assisted ureterocalicostomy for treatment of primary and recurrent pelvi-ureteric junction obstruction in children: a multicenter comparative study with laparoscopic and robot-assisted Anderson-Hynes pyeloplasty

**DOI:** 10.1007/s11255-022-03305-2

**Published:** 2022-07-21

**Authors:** Ciro Esposito, Thomas Blanc, Dariusz Patkowski, Pedro José Lopez, Lorenzo Masieri, Anne-Francoise Spinoit, Maria Escolino

**Affiliations:** 1 Division of Pediatric Surgery Unit, Federico II University Hospital, Via Pansini 5, 80131 Naples, Italy; 2 Department of Pediatric Surgery, Hôpital Universitaire Necker-Enfants Malades, Paris, France; 3 Department of Pediatric Surgery and Urology, Wroclaw Medical University, Wroclaw, Poland; 4 Department of Pediatric Urology, Exequiel González Cortés Hospital, Santiago, Chile; 5 Department of Pediatric Urology, Meyer Children Hospital, Florence, Italy; 6 Department of Urology, ERN Eurogen Accredited Centre, Ghent University Hospital, Ghent, Belgium

**Keywords:** Ureterocalicostomy, Recurrent, PUJO, Robotics, Children, Laparoscopy

## Abstract

**Purpose:**

This multi-institutional study aimed to assess the outcomes of laparoscopic ureterocalicostomy (LUC) and robot-assisted laparoscopic ureterocalicostomy (RALUC) and compare them with laparoscopic pyeloplasty (LP) and robot-assisted laparoscopic pyeloplasty (RALP) in children with pelvi-ureteric junction obstruction (PUJO).

**Methods:**

The data of 130 patients (80 boys), with median age 7.6 years and median weight 33.8 kg, receiving minimally invasive treatment of PUJO over a 6-year period, were retrospectively analyzed. Patients were grouped according to the operative approach: G1 included 15 patients, receiving LUC (*n* = 9) and RALUC (*n* = 6), and G2 included 115 patients, receiving LP (*n* = 30) and RALP (*n* = 85). Patient characteristics and operative outcomes were compared in both groups.

**Results:**

The median patient age and weight were significantly higher in G1 than in G2 [*p* = 0.001]. The median operative time was similar in both groups (157.6 vs 150.1 min) [*p* = 0.66] whereas the median anastomotic time was shorter in G1 than in G2 (59.5 vs 83.1 min) [*p* = 0.03]. The surgical success rate was similar in both groups (100% vs 97.4%) [*p* = 0.33]. Post-operative complications rate was higher in G1 than in G2 (20% vs 6.1%) but all G1 complications were Clavien 2 and did not require re-intervention.

**Conclusion:**

LUC/RALUC can be considered safe and effective alternative approaches to LP/RALP for PUJO repair and reported excellent outcomes as primary and salvage procedures. Robot-assisted technique was the preferred option to treat most patients with recurrent PUJO in both groups.

**Supplementary Information:**

The online version contains supplementary material available at 10.1007/s11255-022-03305-2.

## Introduction

Anderson-Hynes dismembered pyeloplasty represents the most common surgical approach adopted for pelvi-ureteric junction obstruction (PUJO) repair. In recent years, laparoscopic and robot-assisted approaches have become effective alternatives to open technique, providing excellent results, also for management of cases with complex anatomy and previous failed pyeloplasty [1–3].

Ureterocalicostomy (UC) has been described as an alternative technique to Anderson-Hynes dismembered pyeloplasty for management of selected cases, such as giant intra-renal pelvis, PUJO associated with anatomical anomalies such as horseshoe kidney or malrotated kidney or recurrent PUJO with dense scarring making redo pyeloplasty difficult or impossible [4–7].

Initially described by Neuwirt in 1948, UC involves the excision of the hydronephrotic thinned lower pole parenchyma and anastomosis of the dismembered ureter directly to the lower pole calyx to provide effective drainage [8, 9]. At beginning, an open approach was preferentially adopted to perform UC, due to the complexity of this technique, especially in recurrent PUJO [9]. However, open approach was associated with longer operative time and higher morbidity rates due to larger surgical incisions, longer length of stay, and increased analgesic therapy [10–12]. These disadvantages encouraged urologists to explore less invasive surgical options.

Laparoscopic ureterocalicostomy (LUC) was reported as a safe and feasible option in selected PUJO cases with parenchymal thinning due to atypical anatomy or failed pyeloplasty [13]. More recently, robot-assisted laparoscopic ureterocalicostomy (RALUC) has been reported as a viable and technically feasible treatment option for patients with recurrent PUJO or with difficult intra-renal pelvis [7, 14].

The efficacy of UC, as both primary and salvage technique, has been largely demonstrated and both laparoscopic and robot-assisted techniques have been described in the adult literature [15, 16]. Conversely, small case series of UC, using both laparoscopic and robot-assisted approach, have been reported in the pediatric population [17–20].

This multicenter international study aimed to assess the outcomes of LUC and RALUC and compare them to laparoscopic pyeloplasty (LP) and robot-assisted laparoscopic pyeloplasty (RALP) in children with PUJO.

## Materials and methods

The medical charts of 130 patients (80 boys), with median age 7.6 years and median weight 33.8 kg, receiving minimally invasive treatment of PUJO in 6 international pediatric surgery units over a 6-year period (December 2015 to December 2021), were retrospectively analyzed. Specific inclusion criteria were age > 3 years and weight > 20 kg for robot-assisted surgery and age > 1 year and weight > 10 kg for laparoscopic approach. Patients were grouped according to the operative approach: G1 included 15 patients, receiving LUC (*n* = 9) and RALUC (*n* = 6), and G2 included 115 patients, receiving LP (*n* = 30) and RALP (*n* = 85).

Pre-operative work-up included ultrasonography (US) for pelvic antero-posterior diameter (APD), and diuretic renal scan for split renal function (SRF) and drainage.

Our follow-up scheme included renal US at 1–3–6–12 months postoperatively and diuretic renal scan at 1 year postoperatively. Thereafter, patients performed renal US annually for at least 5 years after surgery. The minimal follow-up time in this study was 6 months.

This study received the appropriate Institute Review Board (IRB) approval.

### LUC and RALUC operative technique

**Fig. 1:**
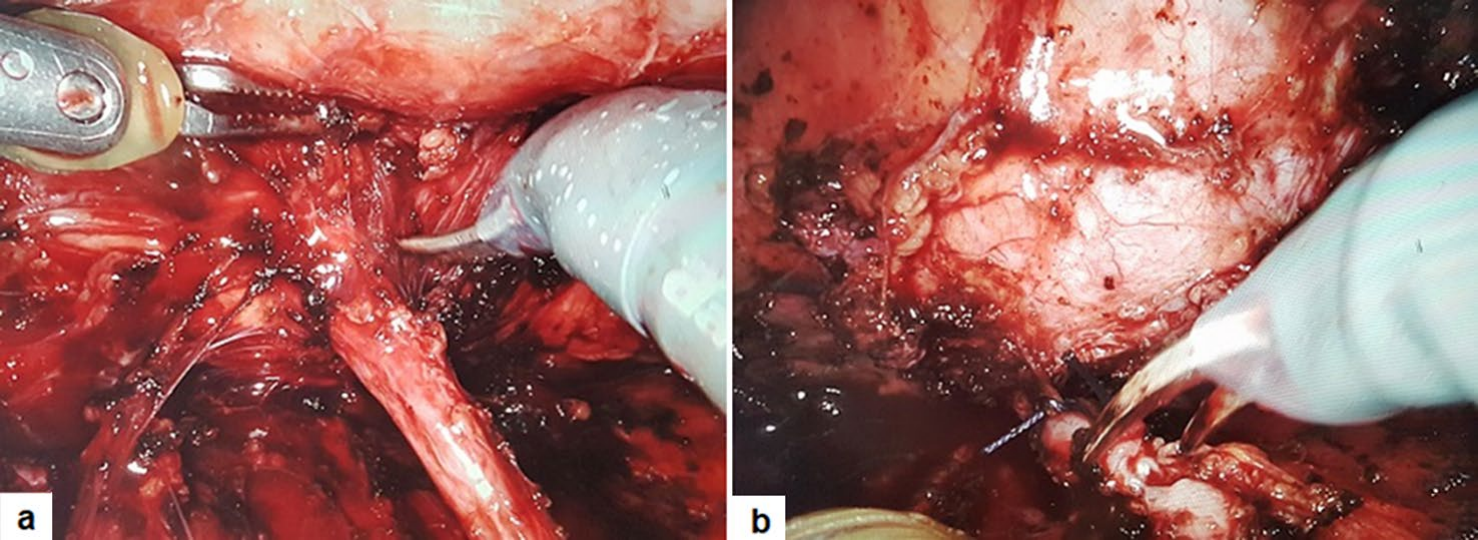
The ureter was mobilized (**a**), ligated and disconnected from the renal pelvis (**b**)

**Fig. 2:**
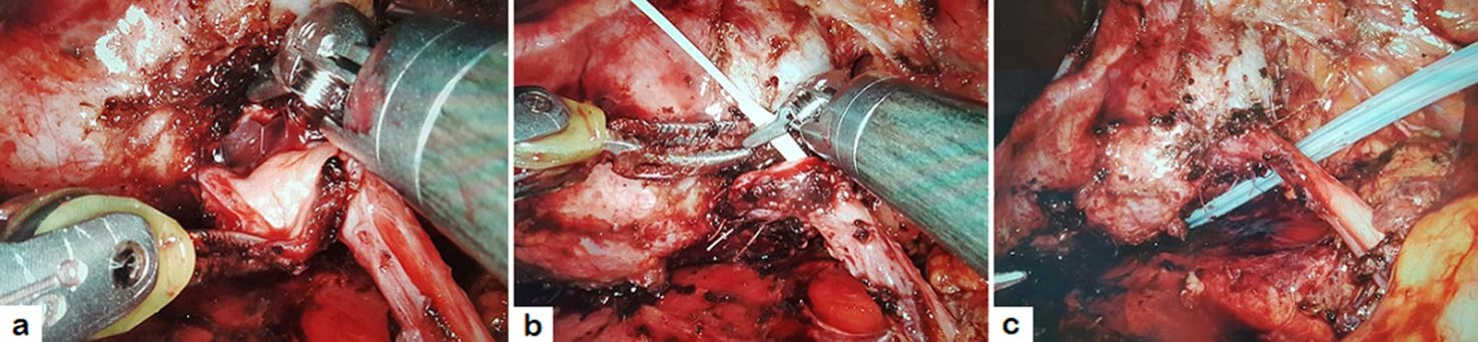
The posterior wall of anastomosis was performed using running suture (**a**); a JJ stent was placed in an antegrade fashion over a guidewire (**b**); a perirenal drain tube was placed (**c**)

All patients underwent minimally invasive (laparoscopic/robot-assisted) transperitoneal UC. Patients were placed in semilateral flank position, with the operative side lifted using a pad underneath. A Foley catheter was placed into the bladder using sterile precautions. Both LUC and RALUC followed the same surgical steps. The colon was detached in all cases to easily expose the dilated kidney. The ureter was mobilized carefully to bring it closer to the lower pole (Fig. 1a). Then, it was ligated with resorbable sutures at the level of the renal pelvis or crossing vessels if the pelvis was not readily accessible and disconnected from the renal pelvis (Fig. 1b). The ureter was spatulated at least 1 cm before anastomosing it with the lower pole calyx. The most dependent part of the lower pole calyx was identified. To ensure a wide anastomosis and minimize the risk of stenosis, the renal parenchyma was largely incised to expose a sizeable area of the most dependent lower pole calyx. A tension-free anastomosis between the spatulated proximal ureter and the opened lower pole calyx was then created. The posterior wall of anastomosis was performed using a 5–0 polyglicolic acid running suture (Fig. 2a). Thereafter, the anterior anastomosis was performed using interrupted stitches, after ensuring that JJ stent was placed in an antegrade fashion over a guidewire introduced through the accessory port (Fig. 2b). At the end of procedure, a perirenal drain tube was placed for at least 24–48 h postoperatively (Fig. 2c). The JJ stent was removed after 4–6 weeks postoperatively.

### Outcomes

Patient characteristics and operative outcomes were compared between both groups. Patient baseline evaluated were age, gender, weight, side of pathology, presence of symptoms. Particular attention was paid to the surgical indications and the technical details of the procedure.

The primary outcome of the study was surgical success rate. This was defined by absence of symptoms and/or decrease of pelvic antero-posterior diameter (APD) on post-operative US and/or improved drainage and/or preserved or improved SRF on post-operative diuretic renogram compared with pre-operative values.

Secondary outcome measures included: total operative time (including access, docking, reconstruction with stenting and closure), anastomotic time, length of stay (LOS), conversions, intra- and post-operative complications and need for re-operations. Post-operative complications were graded according to Clavien Dindo classification [21].

### Statistical analysis

Statistical analysis was carried out using the Statistical Package for Social Sciences (SPSS Inc., Chicago, Illinois, USA), version 13.0. Continuous data were summarized and presented as median with range. The categorical variables were presented as absolute numbers and percentages.

The categorical variables were analyzed using χ2 test and the continuous data were measured using Mann Whitney *U* test. *P* < 0.05 was considered statistically significant.

## Results

The median patient age was 10.1 years (range 3–17) in LUC/RALUC group (G1) and 5.1 years (range 1.6–14) in LP/RALP group (G2). The median patient weight was 41.1 kg (range 15–70) in G1 and 26.5 kg (range 11.2–70) in G2. Symptoms at time of diagnosis were present in 14/15 (93.3%) G1 patients and 68/115 (59.1%) G2 patients.

In all patients, initial surgical approach to PUJO, either primary or recurrent, was to perform dismembered Anderson-Hynes pyeloplasty. The decision to switch from Anderson-Hynes to UC was always made intra-operatively, based on anatomical conditions and technical challenge.

In 7/15 (46.7%) G1 patients, UC was performed as primary procedure for PUJO associated with unfavourable anatomy. This included intra-renal hydronephrosis with minimal or no evident extra-renal pelvis for reconstruction (*n* = 5) and renal malrotation (*n* = 2). In these 7 children, it was judged that conventional dismembered pyeloplasty anastomosis at the level of the renal pelvis could not ensure an adequate dependent drainage. In 8/15 (53.3%) G1 patients, LUC/RALUC was performed as “salvage” procedure for recurrent PUJO after prior failed open Anderson-Hynes dismembered pyeloplasty (Supplementary Table).

LP/RALP was performed for primary PUJO in 99/115 (86.1%) G2 patients. Conversely, LP/RALP was adopted for recurrent PUJO after previous open or laparoscopic dismembered pyeloplasty in 16/115 (13.9%) G2 patients.

The comparative analysis of patient baseline in both groups (Table 1) showed that the median patient age and the median patient weight were significantly higher in G1 than in G2 [*p* = 0.001]. Pre-operative symptoms were more frequent in G1 than in G2 [*p* = 0.001]. Most patients with recurrent PUJO were treated using robot-assisted approach. 5/8 (62.5%) patients with recurrent PUJO received RALUC in G1 and 12/16 (75%) underwent RALP in G2.

**Table 1:** Comparison of patient baseline between LUC/RALUC (G1) and LP/RALP (G2)

Parameter	LUC/RALUC (G1)	LP/RALP (G2)	*P* value
Patient number, *n*	159 (LUC)—6 (RALUC)	115 30 (LP)—85 (RALP)	
Median age, years (range)	10.1 (3–17)	5.1 (1.6–14)	**0.001**
Male/Female, *n*/*n*	11/4	69/46	0.55
Median weight, kg (range)	41.1 (15–70)	26.5 (11.2–70)	**0.001**
Laterality: left/right sid	6/9	66/49	0.33
Asymptomatic, *n* (%)	1 (6.7)	47 (40.9)	**0.001**
Symptoms, *n* (%)	14 (93.3)	68 (59.1)	**0.001**
*UTIs*	*4 (26.6)*	*20 (17.4)*	
*Flank/abdominal pain*	*7 (46.7)*	*36 (31.3)*	
*Combination of pain and UTIs*	*3 (20)*	*12 (10.4)*	
Primary PUJO, *n*	7(6 LUC – 1 RALUC)	99 (26 LP – 73 RALP)	**0.001**
Recurrent PUJO, *n*	8 (3 LUC – 5 RALUC)	16 (4 LP – 12 RALP)	0.66
Pre-operative pelvic APD on US, mm (range)	27.6 (22–38)	29.8 (25–45)	0.68

*LUC* laparoscopic ureterocalicostomy, *RALUC*  robot-assisted laparoscopic ureterocalicostomy, *LP*  laparoscopic pyeloplasty, *RALP*  robot-assisted laparoscopic pyeloplasty, *UTIs*  urinary tract infections, *PUJO*  pelvi-ureteric junction obstruction, *APD* antero-posterior diameter, *US* ultrasonography

The comparative analysis of operative outcomes in both groups (Table 2) showed that the median operative time was similar in both groups (157.6 vs 150.1 min) [*p* = 0.66] whereas the median anastomotic time was significantly shorter in G1 than in G2 (59.5 vs 83.1 min) [*p* = 0.03]. No intra-operative complications or conversions occurred in both groups. The median LOS was also similar in both groups (2.8 vs 2.4 days) [*p* = 0.55].

**Table 2:** Comparison of operative outcomes between LUC/RALUC (G1) and LP/RALP (G2)

Parameter	LUC/RALUC (G1) *n* = 15	LP/RALP (G2) *n* = 115	*P* value
Median operative time, min (range)	157.6 (90–240)	150.1 (100–300)	0.66
Median anastomotic time, min (range)	59.5 (25–95)	83.1 (50–125)	**0.03**
Intra-operative complications, *n* (%)	0	0	
Conversion to open, *n* (%)	0	0	
Median LOS, days (range)	2.8 (2–10)	2.4 (2–5)	0.55
Post-operative complications, *n* (%)	3 urinary leak [Clavien 2] (20)	4 UTIs [Clavien 2] (3.5) 3 anastomosis strictures [Clavien 3b] (2.6)	**0.03**
Re-operations, *n* (%)	0	3 (2.6)	0.35
Median follow-up duration, months (%)	37.2 (6–60)	38.1 (8–63)	0.58
Surgical success, *n* (%)	15 (100)	112 (97.4)	0.33
Resolution of symptoms, *n* (%)	14/14 (100)	68/68 (100)	0.33
*Post-operative US:*			
Improved hydronephrosis, *n* (%)	14 (93.3)	115 (100)	0.66
Worsening hydronephrosis, *n* (%)	1 (6.7)	0	0.35
Post-operative pelvic APD on US, mm (range)	7.4 (5–13)	6.5 (8–14)	0.67
*Post-operative diuretic renogram:*			
Improved drainage, *n* (%)	15 (100)	115 (100)	0.48
Worsening drainage, *n* (%)	0	0	
Preserved SRF, *n* (%)	3 (20)	34 (29.6)	0.56
Improved SRF, *n* (%)	12 (80)	81 (70.4)	0.36
Worsening SRF, *n* (%)	0	0	

*LUC* laparoscopic ureterocalicostomy, *RALUC*  robot-assisted laparoscopic ureterocalicostomy, *LP*  laparoscopic pyeloplasty, *RALP*  robot-assisted laparoscopic pyeloplasty, *LOS*  length of stay, *US*  ultrasonography, *APD*  antero-posterior diameter, *SRF* split renal function

The median length of follow-up was 37.2 months (range 6–60) in G1 and 38.1 months (range 8–63) in G2 [*p* = 0.58]. The surgical success rate was similar in both groups (100% vs 97.4%) [*p* = 0.33]. All patients of both groups were free of symptoms postoperatively. The median pelvic APD, measured on post-operative US, declined significantly in both groups (27.6–7.4 mm in G1; 29.8–6.5 mm in G2) [*p* = 0.001]. Improved drainage and preserved or improved SRF on diuretic renogram, performed at 1 year postoperatively, was observed in all patients of both groups.

Regarding post-operative complications, urinary leak (Clavien 2) occurred in 3/15 (20%) G1 patients, UTIs (Clavien 2) in 4/115 (3.5%) G2 patients and anastomotic stricture (Clavien 3b) in 3/115 (2.6%) G2 patients. Post-operative complications rate was significantly higher in G1 than in G2 [*p* = 0.03] but all complications reported in G1 were Clavien 2 grade and did not require re-intervention.

## Discussion

UC has been described as the operation of choice for the management of most cases of recurrent PUJO as result of scarring and stenosis at the pelvi-ureteric junction (PUJ) preventing reconstruction [2, 22]. Our results confirmed these data: in our series, UC was adopted as “salvage procedure” for recurrent PUJO in 8/15 (53.3%) patients, all of whom reported excellent outcome, with resolution or improvement of hydronephrosis on US and improved drainage on renogram.

In addition to its role as “salvage” procedure, UC may offer distinct advantages over conventional Anderson-Hynes dismembered pyeloplasty also for primary repair of PUJO in selected conditions. A good indication for UC is when PUJO is secondary to complicating anatomical anomalies of the kidney, such as horseshoe kidney or anomalies of renal rotation, a giant intra-renal pelvis or a short ureter [18]. In such anomalies, the aberrant vessels and the anomalous orientation of the renal pelvis and the PUJ cause difficulty in ensuring a valid dependent drainage by conventional pyeloplasty. In 7/15 (46.7%) patients of our series, UC was performed as primary procedure for PUJO associated with intra-renal hydronephrosis and unfavourable anatomy. In these 7 children, we judged that conventional pyeloplasty anastomosis at the level of the renal pelvis could not ensure an adequate dependent drainage. We believe that the anatomical conditions advocating UC in preference to pyeloplasty are presence of thinned cortex overlying dilated lower pole calyx; high insertion of the PUJ; and/or presence of a long proximal segment of stenotic ureter that may compromise tension-free ureteropelvic anastomosis.

Regarding the operative technique, the main steps of minimally invasive approaches (laparoscopic and robot-assisted) for UC did not differ from the open procedure. We believe that, beside the surgical approach adopted, the technical key points for successful UC are: (1) good exposure of lower pole calyx and generous excision of lower pole renal parenchyma overlying the most inferior dependent dilated calyx adjacent to the site of anastomosis; (2) Wide mobilization and spatulation of the ureter to provide a tension-free watertight ureterocaliceal anastomosis; (3) Closure of the renal pelvis at the site of the original PUJ or crossing vessels if the pelvis was not readily accessible. In our experience, the renal pelvis was not reduced in any case and the renal hilum was circumferentially mobilized and visualized. In our hands, this technique proved to be feasible, reporting an overall length of surgery like in LP/RALP but shorter time to complete the anastomosis.

Concerns remain about the outcome of LUC/RALUC. Bleeding from the incised renal parenchyma and the risk of anastomotic stricture and subsequent recurrent obstruction represent the most common complications of UC [23]. In performing LUC/RALUC, control of bleeding from the anastomotic site is one of the most crucial issues [24]. In all patients of our series, the renal parenchyma at the lower calyx was thin enough to incise it without risk of bleeding. The thickness of the renal parenchyma at the anastomotic site represents another key factor in patient selection for LUC/RALUC.

Recurrent obstruction following LUC/RALUC might be caused by scarring at the anastomotic site due to ischemic damage of renal parenchyma or ureter. This complication can be minimized by generous excision of the renal parenchyma at the anastomotic site and a tension-free anastomosis [14]. No patients in our series experienced recurrent obstruction; probably, the trans-anastomotic stenting most likely reduced the urinary extravasation, the formation of perianastomotic fibrosis and subsequent anastomotic stricture.

Our comparative analysis between the outcomes of LUC/RALUC and LP/RALP showed no significant differences with respect to success rate, overall length of surgery and re-operation. Post-operative complications rate was higher in LUC/RALUC series, but the occurred complications were all Clavien 2 and did not require any additional surgery.

Regarding the choice of surgical technique, robot-assisted approach was the preferred option to treat most patients with recurrent PUJO in both groups. The use of robotic technology, providing delicate dissection and fine suturing, reported excellent surgical outcomes also in challenging scenarios such as recurrent PUJO. The additional advantages of using robotics are three-dimensional visualization and increased freedom of movement compared to conventional laparoscopy [25, 26]. Accordingly, RALUC may be considered a promising option in the pediatric population, although the limitations to its widespread adoption remain the high costs, the patient age, and the availability of the robotic platform.

Limitations of this study include the retrospective design, the multi-institutional participation, and the heterogeneity of study groups, not allowing to perform a head-to-head comparison of UC with Anderson-Hynes pyeloplasty. However, given the rarity of this condition, it would be very difficult to perform a well-designed prospective study.

The experience reported in the present study endorsed the role of UC as versatile and reliable procedure for a variety of indications in pediatric patients, such as recurrent PUJO and primary PUJO with unfavourable anatomy. Both LUC and RALUC can be considered safe and effective alternative approaches for PUJO repair in children and reported excellent outcomes as primary and salvage procedures. Robot-assisted approach was the preferred option to treat most patients with recurrent PUJO in both groups.

## Supplementary Information

Below is the link to the electronic supplementary material.

Supplementary file1 (DOCX 18 KB)
