# Supplementary material for: Laparoscopic and robot-assisted ureterocalicostomy for treatment of primary and recurrent pelvi-ureteric junction obstruction in children: a multicenter comparative study with laparoscopic and robot-assisted Anderson-Hynes pyeloplasty
Source: Int Urol Nephrol. 2022 Jul 21;54(10):2503–9. doi: 10.1007/s11255-022-03305-2 (PMC9463286; doi:10.1007/s11255-022-03305-2)
Supplement: Supplementary file 1 — Supplementary file1 (DOCX 18 KB) [file 11255_2022_3305_MOESM1_ESM.docx]

**Supplementary Table:** Characteristics of patients undergoing LUC/RALUC

| **Patient** | **Age (years)** | **Gender** | **Weight (Kg)** | **PUJO Side** | **Surgical Procedure** | **Symptoms** | **Surgical Indication** | **Follow-up (months)** | **Surgical Unit** |
| --- | --- | --- | --- | --- | --- | --- | --- | --- | --- |
| 1 | 3 | F | 15 | R | LUC | YES | Intra-renal hydronephrosis | 55 | 1 |
| 2 | 9 | M | 36 | L | LUC | YES | Intra-renal hydronephrosis | 34 | 1 |
| 3 | 4.5 | M | 24 | R | LUC | YES | Intra-renal hydronephrosis | 28 | 1 |
| 4 | 7 | F | 27 | R | LUC | NO | Intra-renal hydronephrosis | 32 | 1 |
| 5 | 8 | F | 31 | L | LUC | YES | Recurrent PUJO | 6 | 1 |
| 6 | 5 | M | 22 | L | LUC | YES | Intra-renal hydronephrosis | 60 | 2 |
| 7 | 7 | M | 27 | L | LUC | YES | Malrotated kidney | 46 | 2 |
| 8 | 11 | M | 31 | R | LUC | YES | Recurrent PUJO | 60 | 3 |
| 9 | 5 | M | 25 | R | LUC | YES | Recurrent PUJO | 51 | 3 |
| 10 | 16 | F | 52 | R | RALUC | YES | Recurrent PUJO | 36 | 3 |
| 11 | 12 | M | 39 | L | RALUC | YES | Malrotated kidney | 34 | 3 |
| 12 | 17 | M | 65 | R | RALUC | YES | Recurrent PUJO | 28 | 4 |
| 13 | 17 | M | 70 | L | RALUC | YES | Recurrent PUJO | 40 | 5 |
| 14 | 14 | M | 38 | R | RALUC | YES | Recurrent PUJO | 30 | 6 |
| 15 | 16 | M | 52 | R | RALUC | YES | Recurrent PUJO | 18 | 6 |

LUC = laparoscopic ureterocalicostomy; RALUC= robot-assisted laparoscopic ureterocalicostomy; PUJO= pelvi-ureteric junction obstruction; R= right; L= left; M= male; F= female
